# The Mediating Role of Mental Adjustment in the Relationship between Perceived Stress and Depressive Symptoms in Hematological Cancer Patients: A Cross-Sectional Study

**DOI:** 10.1371/journal.pone.0142913

**Published:** 2015-11-20

**Authors:** Yingchun Li, Ying Yang, Rong Zhang, Kun Yao, Zhuogang Liu

**Affiliations:** Department of Hematology, Shengjing Hospital, China Medical University, #39 Huaxiang Road, Shenyang, 110021, China; Xi'an Jiaotong University School of Medicine, CHINA

## Abstract

**Background:**

Depression is a particularly common psychological disorder that affects cancer patients. Diagnosed with hematological malignancies constitute a serious unpredictable and uncontrollable medical stress situation and patients are susceptible to suffer from depressive symptoms. The aims of the study were to explore the correlation between perceived stress and depressive symptoms in patients with hematological malignancies, and assess the mediating role of mental adjustment between these variables.

**Methods:**

A single center, cross-sectional study was performed by convenience sampling between July 2013 and April 2014 in a hospital of China. The Center for Epidemiologic Studies Depression Scale, Perceived Stress Scale, and Mini-Mental Adjustment Scale, as well as questions about demographic and clinical factors was distributed to 300 hematological cancer patients. Completed questionnaires were received from 227 inpatients.

**Results:**

The results showed that perceived stress was positively correlated with depressive symptoms. The mental adjustment significantly mediated the relationship between perceived stress and depressive symptoms.

**Conclusions:**

Among hematological cancer patients perceived stress may be a risk factor for depressive symptoms, whereas positive coping style might be protective against depressive symptoms. Results showed that medical managers could support the development of mental adjustment in the patients to alleviate psychological disorders.

## Introduction

Depression is a particularly common psychological disorder among cancer patients, has a major impact on sleep disturbances, fatigue, treatment adherence, quality of life and cancer survival [1–4]. Prevalence estimates of persistent depression in cancer patients ranged from 17.6% to 66.72% [5, 6]. More than 850,000 patients were diagnosed with a hematological cancer each year all over the world [7]. Hematological cancers consist of numerous subtypes, from aggressive to chronic, and each subtype differs in its characteristics and rate of progression. Hematological cancer patients always require constant surveillance with intermittent regimens of treatment. Different from solid tumor, the treatment regimens for hematological malignancies are multiple, which include high-dose chemotherapy or hematopoietic stem cell transplantation, and often urgent, lengthy and aggressive [8–11]. Previously reported results suggested that depressive symptoms could be explained largely by psychosocial (such as age/gender/ income) and its disease factors (such as types/course/grade of disease) among cancer patients [12–14]. However, these factors cannot be intervened in by patients themselves or their medical staff. Meanwhile, the psychological response to this life-threatening disease has not been received enough attention compared with other malignancies, nor are the standards for psychological care in this circumstance well established.

Research has consistently reported that psychosomatic symptoms are caused by stress in neuroscience, biology and psychology [15]. Stress can be considered as the state in which environmental demands or mental strain exceed a person’s regulatory capacity [16].It is reported that acute and chronic stressful life events in early life had substantial influence on the later development of depression [17, 18]. Hematological malignancy is an uncontrollable event, and the treatment outcomes are always unpredictable. This process are risk factors triggering a severe psychological stress, which derives from uncertainty of the future, the sense of life threat, and negative psychological changes [19].

According to the transactional model [20, 21], the process of dealing with stress consists of independent, mediating and outcome variables. Environmental and personality variables, such as the levels of individual’s perceived stress, are causal antecedents of the adaptation to stress events; the individual’s more or less successful adaptation to stress is the outcome. The effect of these independent variables is mediated by the individual’s coping and appraisal. ‘Mental adjustment’ to cancer refers to a patient’s cognitive and behavioral responses to a cancer diagnosis [22]. According to cognitive theory, patients develop depression not only because they have cancer, but also because of how they perceive and interpret their situations [23]. In patients with leukemia and lymphoma, a worse coping style was correlation with severe psychological distress. As demonstrated by Montgomery et al., patients with low scores on the fighting spirit had adjusted poorly to their cancer diagnosis [24]. Some studies also reported that, in women with breast cancer, fighting spirit was found to be the primary coping style used in the non-depression group, while helplessness/hopelessness, anxious/preoccupation and fatalism were the coping styles used the most in the depression group [25, 26].Otherwise, mental adjustment are considered to be probable mediators of the relations between self-efficacy and well-being in cancer patients [27] and have also been found to mediate the impact of other personalities, such as trait pessimism and age, on psychological adjustment [28, 29].

The available literatures identify several variables that directly impact the depressive symptoms by psychosocial factors of individuals with malignant tumor. However, few of studies analyze how different factors mediate the relationship between these variables exist. This leaves a significant gap in the current research base. Analysis of how mental adjustment may mediate the relationship between perceived stress and depressive symptoms, and the actual experience of psychological distress is of great importance to advance the knowledge base and understanding of depressive symptoms for patients with hematological cancer. We have studied 227 hematological cancer patients who were being hospitalized to investigate whether perceived stress correlates with depressive symptoms and whether adjustment styles mediate this association, in a cross-sectional design.

## Materials and Methods

### Participants

The questionnaires were collected from July 2013 to April 2014 in the department of hematology from Shengjing Hospital of China Medical University. This study was approved from the Committee on Human Experimentation of Shengjing Hospital of China Medical University. Eligible patients who agreed to participate in this cross-sectional study were given an informed consent paper to read and sign. Eligibility criteria concluded that patients a) were at least 18 years old, b) had been clinically diagnosed with hematological cancers, c) have been known their diagnoses, d) were native Chinese speakers, e) have cognitive competence. Patients who were excluded were as follows: patients a) had a history of psychiatric disorders, b) were illiterate to complete the survey, c) had other hematological system diseases or other cancers. Finally, we recruited 300 eligible hematological cancer inpatients into the study and 227 questionnaires were included in analysis. The effective response rate was 75.67%.

### Measures

Demographic and clinical variables were completed by a general questionnaire. Demographic variables included age, gender, marriage, education level, income level, residence and payment. Clinical variables were collected by attending doctors according to the medical records such as diagnosis time, disease type, chemotherapy phase, and so on.

Depressive symptoms were assessed by the Center for Epidemiologic Studies Depression Scale (CES-D) [30]. The scale consisted of 20 questions and each question was scored on a 4-point Likert scale ranging from “rarely or none of the time” (0 points) to “most or all of the time” (3 points). The total score ranged from 0 to 60. “Depressive symptoms” were defined as subjects score was of 16 or more. In this study, the Cronbach’s alpha of the CES-D scale was 0.890.

Perceived stress was measured with Perceived Stress Scale-10 (PSS-10) [31]. The PSS-10 was designed to measure the level of perceived aspects of one’s life which was uncontrollable, overloading, and unpredictable. Each question was on a 5-point Likert scale ranging from 0 (never) to 4 (very often).The total score could range from 0 to 40. Higher composite scores indicated greater perceived stress. Score of 20 or higher was considered high stress. Chinese version of the scale has also demonstrated good reliability and validity [32]. The PSS-10 has demonstrated good reliability in this study (Cronbach’s alphas = 0.768).

Mental adjustment was assessed with the Mini-Mental Adjustment to Cancer (Mini-MAC) scale which was developed to measure cognitive and behavioral responses of patients suffering from cancer [33]. It consisted of 29-item with five adjustment styles: Fighting Spirit, Helplessness/Hopelessness, Anxious Preoccupation, Fatalistic, and Avoidance. Chinese Mini-MAC version was mostly considered as three factors structure which could be described as Negative Emotion, Positive Attitude, and Cognitive Avoidance, respectively [34]. In our study, three subscales of the questionnaire showed good reliability (Negative Emotion: Cronbach’s alphas = 0.940, Positive Attitude: Cronbach’s alphas = 0.763, Cognitive Avoidance: Cronbach’s alphas = 0.749).

### Statistical methods

We used frequency statistics and t-test or one-way ANOVA to describe distributions of depressive symptoms in categorical demographic and clinical variables. Continuous variables correlation was calculated by Pearson’s correlation analyses. Hierarchical linear regression and asymptotic and resampling strategies were used to test the main study hypotheses. For example, in step1, participants’ demographic and clinical information which were significantly differences in depressive symptoms tested by ANOVA or Pearson’s correlation would be entered as control variables. In step 2, perceived stress was entered if it was correlated with dependents. In step3, negative emotion, positive attitude, and cognitive avoidance which were correlated with dependents were entered. Output results including R^2^, adjust R^2^(Adj.R^2^), R^2^- changes, F value and standardization regression coefficient(β) were provided in the regression models. All study variables were standardized before hierarchical linear regressions. Asymptotic and resampling strategies were performed to examine the mediation effect which was an increasingly popular non-parametric method of testing mediation effect [35]. In the equations, CES-D score was modeled as the dependent variable, perceived stress as the independent variable, and the subscales of MAC as the mediators. Significant demographic or clinical information such as age was as covariates. The bootstrap estimate presented in our study was based upon 1,000 bootstrap samples. Analyses were conducted using SPSS for Windows (version 17.0) and a *p* value of 0.05 was set as the level of statistical significance (two-tailed).

## Results

### Characteristics of subjects

Table 1 showed demographic and clinical characteristics of participants. Average age was 45.48 years old (SD = 16.05), ranging from 18 to 83. The longest course of disease was more than 12 years and the shortest was less than 1 month since diagnosed (median = 4 months). In this study, based on the cut-off values, the overall prevalence of depressive symptoms were 66.1%. The mean values were(20.18±9.65) for depressive symptoms. Independent sample t-test or one-way ANOVA indicated that there were no statistically significant relationships between depressive symptoms and categorical demographic variables, as well as clinical variables (*P*>0.05).

**Table 1:** Descriptive information on demographic and clinical characteristics.

		N	%	depression	
				M	SD
**Demographic variables**					
gender	male	117	51.5	20.5	9.7
	female	110	48.5	19.9	9.6
marriage	single	38	16.7	21.8	9.6
	Married/cohabitation	179	78.9	20.0	9.7
	Separated/divorced/widow	10	4.4	18.0	9.6
education	Junior middle school or the following	108	47.6	21.5	10.0
	High or secondary school graduated	74	32.6	18.5	9.5
	Bachelor or above	45	19.8	19.7	9.0
Monthly income(yuan)	<1000	88	38.8	21.0	10.0
	1001–2000	66	29.1	19.5	9.5
	2001–3000	37	16.3	20.4	8.8
	>3000	36	15.9	19.1	10.2
Medical payment	Part of public	174	76.7	19.9	9.7
	Medical insurance/self-payment	53	23.2	21.0	9.5
**Clinical variables**					
Disease type	leukemia	149	65.6	20.8	9.4
	Multiple myeloma	28	12.3	19.2	9.2
	lymphoma	50	22.0	18.8	10.6
Disease status	New onset	49	21.6	19.2	10.4
	Relapse/treatment failure	59	26.0	22.0	9.4
	Maintenance treatment	119	52.4	19.7	9.5
Phase of chemotherapy	Not received chemotherapy	7	3.1	20.4	7.4
	Inductive therapy	82	36.1	19.3	10.3
	Consolidation therapy	110	48.5	20.8	9.0
	Maintain intensive therapy	28	12.3	20.4	11.0
**Psychological variables score**	Mean(SD)				
Perceived stress	18.5(5.2)	0	33		
Negative emotion	33.8(10.4)	16	64		
Positive attitude	26.9(4.2)	13	36		
Cognitive avoidance	11.6(2.4)	4	16		
Depressive symptoms	20.2(9.7)	1	45		

M: Mean; SD: Standard deviation.

### Correlations among continuous variables

Correlations among continuous variables were listed in Table 2. Depressive symptoms were positively correlated with perceived stress (γ = 0.668, p<0.01), negative emotion (γ = 0.590, p<0.01) and negatively correlated with age (γ = -0.156, p<0.01), positive attitude score (γ = -0.385, p<0.01), and cognitive avoidance (γ = -0.187, p<0.01). There was no significant correlation between course of the disease and depressive symptoms (γ = -0.034, p>0.05).

**Table 2:** Correlations among Major Variables.

	1	2	3	4	5	6	7
1.age	1						
2.time since diagnosis	.035	1					
3.perceived stress	-.150*	.040	1				
4.negative emotion	-.041	-.011	.488**	1			
5.positive attitude	.067	.103	-.290**	-.083	1		
6.cognitive avoidance	-.063	.054	-.081	.091	.571**	1	
7.depressive symptoms	-.156*	-.034	.668**	.590**	-.385**	-.187**	1

*p <0.05

**p <0.01

### Hierarchical regression analyses

Independent variables which were related to depressive symptoms in univariate analyses were entered into the hierarchical multiple regression models with adjustment for age. As showed in Table 3, perceived stress was positively associated with depressive symptoms in Step 2. In step 3, negative emotion was significantly and positively associated with depressive symptoms and positive attitude was significantly and negatively associated with depressive symptoms. In addition, the effect of perceived stress on depressive symptoms in step 3 was reduced compared with that in step 2, as indicated by smaller β coefficients.

**Table 3:** Hierarchical regression analysis results.

	Depressive symptoms					
	Step 1		Step 2		Step 3	
	β	95%CI	β	95%CI	β	95%CI
age	-0.156*	(-0.018, -0.002)	-0.057	(-0.010, 0.003)	-0.073	(-0.010, 0.001)
stress			0.659***	(0.560, 0.758)	0.413***	(0.311, 0.515)
Negative emotion					0.379***	(0.282, 0.477)
Positive attitude					-0.176**	(-0.283, -0.068)
Cognitive avoidance					-0.093	(-0.198, 0.012)
F	5.621*		91.251***		65.233***	
R^2^	0.024		0.449		0.596	
adjR^2^	0.020		0.444		0.587	
R^2^-change	0.024		0.425		0.147	

*p <0.05

**p <0.01

***p <0.001; 95%CI: 95% confidence interval of difference

### Mediating effects of psychological adjustment variables

Path coefficients a (perceived stress to negative emotion, positive attitude and cognitive avoidance) and b (direct effects of negative emotion, positive attitude and cognitive avoidance on depressive symptoms), a*b products, BCa95%CI for these products, coefficients c (total effects of perceived stress on depressive symptoms) and c’ (direct effects of perceived stress on depressive symptoms) were presented in Table 4. For the model, perceived stress was associated with negative emotion, positive attitude and cognitive avoidance. Negative emotion and positive attitude were significantly associated with depressive symptoms after controlling for age. They were consistent with the results from hierarchical multiple regressions. Thus, significant mediating effects of negative emotion (a*b = 0.1870, BCa95%CI: 0.1244, 0.2561), positive attitude (a*b = 0.0086, BCa95%CI: 0.0177, 0.0978) on the association between perceived stress and depressive symptoms were demonstrated. The proportion of total effect of perceived stress on depressive symptoms by mediator role was calculated with the formula “(a*b)/total effect “. The proportions of mediating roles of negative emotion and positive attitude were 28.4% and 7.6%, respectively.

**Table 4:** Mediating roles of psychological adjustment on the depressive symptoms.

Mediators	a	b	a*b	BCa95%CI	c	c’
Negative emotion	0.4929***	0.3794***	0.1870*	(0.1244,0.2561)	0.6590***	0.4131***
Positive attitude	-0.2862***	-0.1756**	0.0503*	(0.0177,0.0978)		
Cognitive avoidance	-0.0926***	-0.0928	0.0086	(-0.0044,0.0339)		

BCa95%CI: the bias-corrected and accelerated 95% confidence interval

a: associations of Perceived stress with psychological adjustment

b:associations of psychological adjustment with depressive symptoms after controlling for the predictor variables

a*b: the product of a and b

age was covariate.

***p<0.001

**p<0.01

*p<0.05

## Discussion

The present study explored the associations between perceived stress, mental adjustment, and depressive symptoms, and further examined the mediating role of mental adjustment. Depression, the psychological state, is more often correlated with cancer. The prevalence of depressive symptoms was 66.1% in this sample group, which was similar to that reported in Chinese cancer patients (66.72%), and higher than Chinese general population sample (33.3%) [5]. Present results supported the assertion that hematological cancer patients might suffer from high rates of depressive symptoms. It is possible that patients with hematological malignancies always suffers from a difficult treatment process and confront the possibility of the disease recurrence [36]. The social and economic factors also make patients perceive great stress, such as poor marital status, unsustainable cost of treatment, and educational attainment [37, 38]. Patients may unable to cope with the continuously strain, associate with more symptoms of psychological disorders, and fall in to a state of depression. The results in this study suggest that there is a need for medical or nursing managers, social workers and psychologists to develop and implement effective measures to alleviate patients’ depressive symptoms and improve their mental health.

Additionally, our results found that perceived stress was positively correlated with depressive symptoms, which was consistent with previous findings [39]. Psychological stress has been identified as a risk factor for psychosomatic symptoms as it may influence psychological health, quality of life, sleep quality, and physical symptoms [38–42]. A meta-analysis study has shown that high levels of stress were associated with poorer survival, and high cancer mortality [43]. In this study, the score of the perceived stress scale was similar to breast cancer patients who were after surgery [39]. And 47.6% of patients were considered as experiencing high stress, which perceived stress score was above 20. If individuals were in this range, they might consider learning some stress reduction techniques [44]. Various reasons have been suggested for the experience of mental distress. In addition to the cancer itself as a source of mental stress in cancer patients, the symptoms burden of patients, fear of cancer recurrence, and even quality of life are also among the other contributions to stress [36, 41, 45, 46]. It is the fact that patients who were suffering from cancers feel themselves having a need to cope with the challenge events. Different strategies used to cope with stressful situations would result in different mental outcomes [45]. Our results suggested that perceived stress associated with mental adjustment, which motivated us to explore the indirect effect of perceived stress in predicting depressive symptoms. Otherwise, the results encourage further research and development of preventative care to alleviate perceived stress and depressive symptoms among hematological cancer patients.

Coping style is an important resource of psychological adjustment for cancer patients to combat destructive emotions and stress. Consistent with this, in the present study negative emotions was found to be positively associated with depressive symptoms among hematological cancer patients, and positive attitude was negatively associated with depressive symptoms. Patients with high levels of positive attitude to disease might have more expectation and confidence in adapting to adverse challenge and vice versa [47]. Similar results also found in another research. It was reported that patients who received high score on emotional approaching coping showed an increase in well-being over time [48].

As major part of this research an indirect pathway model was developed to determine if mental adjustment significantly mediated the relationship between perceived stress and depressive symptoms in hematological cancer patients. A multiple regression analysis revealed that mental adjustment partially mediated the relationship between perceived stress and depressive symptoms for patients in the study. In other words, mental adjustment serves as a filter through which perceived stress passes, partially determining whether or not patients will experience depressive symptoms related to the nature of their perceived the level of stress. In practice, this result indicated that mental adjustment partially accounts for the relationship between levels of stress experienced by patients. Importantly, there is a great need for medical or psychological researchers to develop necessary approach to help individuals improve their psychological adjustment, to reduce psychological distress and improve quality of life among hematological cancer patients.

The findings have theoretical and practical implications for health care management. In theory, this study lends to support to the mediated pathway model for analyzing relationships between perceived stress and depressive symptoms for patients with hematological malignancies. And provide theoretical insight into explaining a significant gap that exists in the literature and provides a solid foundation for future research in this area. Practically, our findings highlight that hematological cancer patients are experiencing a high level of depressive symptoms and psychological stress. There is an urgent need for them to explore psychological interventions to encourage patients combating psychological distress by a more positive and active coping style [49]. For example, Silva et al. reported that provide individuals more social support would make them tend to express their negative thoughts and emotions, this self-disclosure induced patients to cognitively process the cancer experience [50].

However, several limitations of this research must be taken into consideration when interpreting the results. First, self-report of participants might lead to response bias from negative affect. Second, the study only explored associations between perceived stress, mental adjustment and depressive symptoms, and examined differences in basic demographic and clinical variables. Other factors associated with depressive symptoms should be investigated in future study. Third, because of the cross-sectional study, we are unable to draw conclusions about cause and impact. Our cross-sectional design was that mental adjustment accounted for a part of the predictive association between perceived stress and depressive symptoms, which was consistent with but not confirm a meditational model. A longitudinal design could be considered to increase the power of the findings. Fourth, our participants came from one treatment center and patients were selected by convenience sampling, so that it may limit the representation of the study.

## Conclusions

In our sample, hematological cancer patients suffered from high levels of stress and depressive symptoms. Both perceived stress and mental adjustment were correlated with depressive symptoms and mental adjustment partially mediated the effect of perceived stress on depressive symptoms. Therefore, developed intervention strategies to help patients decrease their depressive symptoms and adopt the right way to cope the stress situation were needed in hematological cancer patients.

## Supporting Information

S1 Table Data of hematological caners. Survey database.
(ZIP)
